# Functional characterization of the human Cdk10/Cyclin Q complex

**DOI:** 10.1098/rsob.210381

**Published:** 2022-03-16

**Authors:** Robert Düster, Yanlong Ji, Kuan-Ting Pan, Henning Urlaub, Matthias Geyer

**Affiliations:** ^1^ Institute of Structural Biology, University of Bonn, Venusberg-Campus 1, 53127 Bonn, Germany; ^2^ Max Planck Institute for Multidisciplinary Sciences, Bioanalytical Mass Spectrometry, 37077 Göttingen, Germany; ^3^ Hematology/Oncology, Department of Medicine II, Johann Wolfgang Goethe University, 60590 Frankfurt am Main, Germany; ^4^ Frankfurt Cancer Institute, Goethe University, 60596 Frankfurt am Main, Germany; ^5^ Institute of Clinical Chemistry, Bioanalytics Group, University Medical Center Göttingen, Göttingen, Germany

**Keywords:** transcription, cell cycle, CDK10, Cyclin Q, Cyclin M, RNA polymerase II

## Abstract

Cyclin-dependent kinases (CDKs) are key players in cell cycle regulation and transcription. The CDK-family member Cdk10 is important for neural development and can act as a tumour suppressor, but the underlying molecular mechanisms are largely unknown. Here, we provide an in-depth analysis of Cdk10 substrate specificity and function. Using recombinant Cdk10/CycQ protein complexes, we characterize RNA pol II CTD, c-MYC and RB1 as *in vitro* protein substrates. Using an analogue-sensitive mutant kinase, we identify 89 different Cdk10 phosphosites in HEK cells originating from 66 different proteins. Among these, proteins involved in cell cycle, translation, stress response, growth signalling, as well as rRNA, and mRNA transcriptional regulation, are found. Of a set of pan-selective CDK- and Cdk9-specific inhibitors tested, all inhibited Cdk10/CycQ at least five times weaker than their proposed target kinases. We also identify Cdk10 as an *in vitro* substrate of Cdk1 and Cdk5 at multiple sites, allowing for a potential cross-talk between these CDKs. With this functional characterization, Cdk10 adopts a hybrid position in both cell cycle and transcriptional regulation.

## Graphical abstract

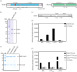

## 1. Introduction

Cyclin-dependent kinases (CDKs) are serine/threonine-specific protein kinases that get activated upon association of a regulatory cyclin subunit. The family of mammalian CDKs comprises 21 different members, which are involved in cell division and transcription [1]. By contrast to the well-established roles of CDKs 1, 2, 4 and 6 in cell cycle regulation, and the emerging picture of CDKs 7, 8, 9 and 12 in transcriptional regulation, other members of the CDK family still await functional investigation.

Although it was discovered 25 years ago, little is known about cyclin-dependent kinase 10 (Cdk10) and its cellular functions. Cdk10 (PISSLRE) is located on chromosome 16 [2]. The Cdk10 gene contains 10 exons and was independently identified in 1994 by two groups upon PCR-based screens attempting to discover cdc2-related protein kinases in human cells [3,4]. The absence of a yeast homologue of Cdk10 has complicated early investigations on Cdk10 cellular functions. In 2013, Cyclin Q (originally named Cyclin M) was identified in a yeast two-hybrid screen as the cognate cyclin partner of Cdk10 [5]. Cyclin Q mutations have been characterized in a rare X-linked gonosomal disease called STAR syndrome [6]. In addition to Cyclin Q, one study describes the association of Cdk10 with Cyclin G2 [7]. Initially, two Cdk10 isoforms have been described: a full-length Cdk10 isoform of 360 amino acids and another N-terminally truncated and C-terminally modified gene product of 316 amino acids [4]. Further analysis revealed additional isoforms and complex splicing of the *CDK10* gene [8,9]. It has been suggested that the isoforms account for different functions and subcellular localization, but their expression and functionality at the protein level has not been systematically addressed. Moreover, data from a yeast two-hybrid screen suggested that the initially identified and truncated isoform 2 is not able to interact with Cyclin Q and thus is very unlikely to form a functional kinase-cyclin complex [5]. Cdk10 expression appears to be stable in dividing and serum-starved cells with a peaking expression upon serum re-stimulation [9]. Another study suggested a specific function of Cdk10 in G2/M phase of the cell cycle [10].

The closely related kinase Cdk11 was found to be important for splicing and the expression of histone genes [11,12], whereas Cdk12 plays a selective role in the expression of DNA damage response genes [13,14]. The multiple stages of transcription regulation controlled by CDKs have made them a promising target for cancer therapy [15]. The role of Cdk10 instead is not yet well defined. Splice-site mutations of Cdk10 lead to developmental defects in humans, and genetic knock-out of Cdk10 in mice is lethal during embryogenesis. However, Cdk10 is dispensable for the maintenance of Cdk10^−/−^ mouse embryonal fibroblasts acquired from these embryos [16]. Furthermore, morpholino-based studies in zebrafish revealed deficits in brain development underlining a developmental function of Cdk10 [17]. Cdk10 has tumour suppressive functions in many cancer types, including breast cancer, gastric carcinoma, liver cancer and glioma, but can also act as an oncogene in colorectal cancer [18]. The tumour suppressive role of Cdk10 in breast cancer was substantiated by a recent study which found that the Cdk10 gene was deleted in 80% of the cases in a Swedish breast cancer cohort [19]. The molecular mechanisms by which Cdk10 suppresses tumour growth are not well understood to date. The best-studied regulatory function is the promotion of proteasomal degradation of the transcription factor ETS-2 by Cdk10-mediated phosphorylation [5,20,21]. The increase in ETS-2 protein upon low Cdk10 expression levels results in increased MAPK signalling and activation of the Raf/Ras pathway, which has been shown to confer tamoxifen resistance in breast cancer [21]. Cdk10 itself can be regulated in breast cancer cell lines by proteasomal degradation, which is promoted by phosphorylation at Thr133 through a yet unidentified kinase [22].

Apart from ETS-2, Cdk10 was found to phosphorylate and thereby downregulate the activity of the PKC-related kinase PKN2, which interacts with Rho/Rac GTPases [23]. The resulting alterations in actin cytoskeleton organization are found to affect the formation of the primary cilia. As a consequence, Cdk10 deficient cells possess elongated cilia upon serum starvation [23]. Besides ETS-2 and PKN2, no other cellular substrates of Cdk10 have been identified until now, illuminating how little is currently understood about the role of Cdk10 in physiologic and pathologic conditions. It is therefore not surprising that Cdk10 was denoted as an understudied kinase [24]. In this study, we purified recombinant Cdk10/CycQ from baculovirus infected insect cells to homogeneity and characterized its ability to phosphorylate known CDK substrates *in vitro*. Moreover, we used a chemical genetic screen to identify native protein substrates of Cdk10 by mass spectrometry directly from cell lysates, taking the first step towards a broader understanding of Cdk10 function.

## 2. Results

### 2.1. Cdk10/CycQ phosphorylates known CDK substrates and is activated by phosphorylation at Thr196

**Figure 1.**
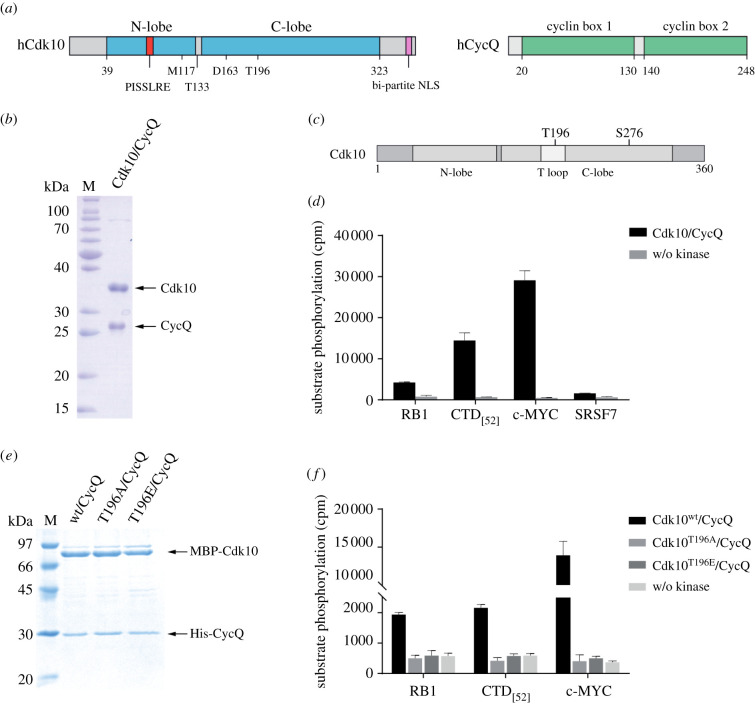
Cdk10 shares substrates with cell cycle and transcriptional CDKs. (*a*) Domain architectures of human Cdk10 (UniProt accession number Q15131) and human Cyclin Q (Q8N1B3). M117—gatekeeper methionine, T133—phosphorylation site, results in increased Cdk10 degradation, D163—catalytic centre, T196—conserved T-loop threonine phosphorylation is required for kinase activation. (*b*) Coomassie-stained SDS–PAGE analysis of 1 µg recombinant human Cdk10/CycQ. (*c*) Mass spectrometry-based detection of phosphorylation within recombinant human Cdk10 expressed in *Sf9* insect cells. (*d*) Radiometric kinase assay; 0.2 µM Cdk10/CycQ, 25 µM GST-RB1 (761–928), 10 µM GST-CTD_[52]_, 25 µM His-c-MYC (17–167) or 25 µM GST-SRSF7 (1–248) were incubated with 1 mM ATP containing 0.35 µCi γ[^32^P]-ATP for 30 min at 30°C. Data represent mean ± s.d. of duplicate measurements; cpm, counts per minute. (*e*) SDS–PAGE analysis (Coomassie) of 2 µg recombinant human MBP-Cdk10/His-CycQ, MBP-Cdk10T196A/CycQ and MBP-Cdk10T196E/CycQ. (*f*) Radiometric kinase assay of Cdk10 T-loop mutants. The experiments were performed similarly as in (*d*).

Human Cdk10 (UniProt accession number Q15131) is a protein of 360 amino acids in length. Unlike Cdk11, 12 and 13, it does not contain large N- or C-terminal sequence extensions in addition to the kinase domain. The kinase domain of Cdk10 is most closely related to Cdk11, sharing a sequence identity of 53% in humans over 330 residues, corresponding to 82% sequence similarity. Within the kinase domain, it contains the PISSLRE sequence, a variation of the Cdk1 PSTAIRE αC helix that is a characteristic Cdk-cyclin interaction motif (figure 1*a*). Kinase activation requires phosphorylation within the activation loop (T-loop) at the conserved Thr196. At the C-terminus, a bi-partite nuclear localization signal is found. Human Cyclin Q (CycQ, UniProt accession number Q8N1B3; also known as Cyclin M or Fam58a of the gene *CCNQ*) is composed of 248 amino acids and contains two cyclin boxes (figure 1*a*). Cyclin Q does not contain a nuclear localization signal, implying that transport into the nucleus is likely to be dependent on Cdk10 interaction or other binding partners.

We expressed the human full-length Cdk10/CycQ complex in *Sf9* insect cells, eluting as stoichiometric heterodimer, and purified the protein complex to homogeneity (figure 1*b*). Mass spectrometry analysis of the protein revealed that Cdk10 is phosphorylated at Thr196 and Ser276 (figure 1*c*). We tested the activity of our kinase preparation towards the four protein substrates retinoblastoma-associated protein (RB1), RNA polymerase II CTD, c-MYC and SRSF7 using recombinant proteins in a radioactive kinase assay (figure 1*d*; electronic supplementary material, figure S1). RB1 is a substrate of the cell cycle CDKs Cdk1, 2, 4 and 6. The RNA pol II CTD is a common substrate of transcription related CDKs, whereas c-MYC has been identified as an *in vitro* substrate for several transcription kinases in our laboratory. SRSF7 has been described as a substrate of Cdk11 [12], which shares the highest sequence similarity with Cdk10 among all CDKs. Cdk10/CycQ phosphorylates RB1, human GST-CTD_[52]_ and c-MYC, though with varying efficacy. SRSF7 instead appears to be a poor substrate for Cdk10 *in vitro*. From these phosphorylation preferences, we conclude that Cdk10 shares substrate proteins with cell cycle and transcriptional CDKs *in vitro*.

CDKs differ in their requirement for T-loop phosphorylation for interaction with their corresponding cyclin subunit. Whereas Cdk7 Thr170 phosphorylation is critical for Cdk7 binding to CycH in the absence of the scaffolding protein MAT1 [25,26], Cdk2 can form inactive CDK-cyclin complexes with CycA in the absence of T-loop phosphorylation [27]. We addressed the requirement of Cdk10 Thr196 phosphorylation for Cyclin Q interaction by mutating Cdk10 Thr196 to either alanine or glutamate. Using MBP-Cdk10 as the bait in co-purification experiments, both Cdk10 (T196A) and Cdk10 (T196E) mutants were able to associate with CycQ demonstrating that the Cdk10–CycQ interaction does not require T-loop phosphorylation (figure 1*e*). However, when tested in kinase activity assays, neither the Cdk10(T196A)/CycQ nor the phosphorylation mimicking Cdk10(T196E)/CycQ complex displayed any activity in contrast to wild-type Cdk10/CycQ (figure 1*f*). This suggests that T-loop phosphorylation is dispensable for Cdk10/CycQ interaction but critical for kinase activation. Of note, the three arginines identified in Cdk2 and other CDKs to coordinate the phosphorylated T-loop [28] are also conserved in Cdk10, contained in the ^80^PISSLREI αC helix, the ^161^HRD catalytic site and the ^181^DFGLAR substrate interaction motif.

### 2.2. Cdk10 phosphorylates serines of RNA pol II CTD with a preference for K7 repeats

**Figure 2.**
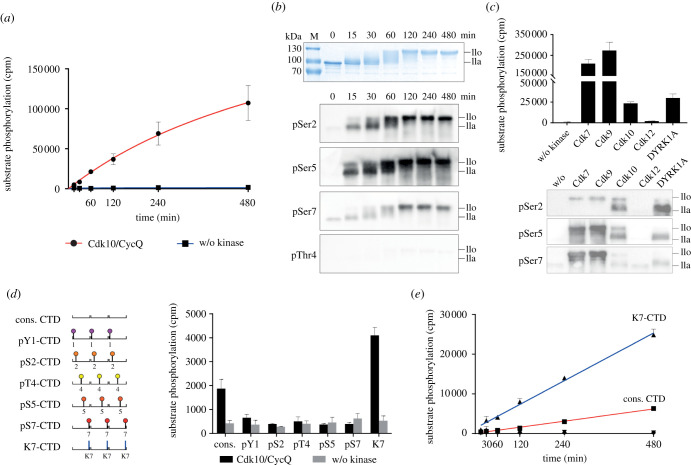
RNA pol II CTD phosphorylation by Cdk10. (*a*) Time course of a radiometric kinase assay. Incubation of 10 µM GST-CTD_[52]_, 1 mM ATP containing 0.35 µCi γ[^32^P]-ATP with or without 0.2 µM Cdk10/CycQ (mean ± s.d. of duplicate measurements; cpm, counts per minute). (*b*) SDS–PAGE analysis (Coomassie) of 2 µg (top panel) and immunoblot analysis (lower panels) of 100 ng GST-CTD_[52]_ after phosphorylation by Cdk10/CycQ. (*c*) Radiometric kinase assay (top panel) and immunoblot analysis (lower panels) of GST-CTD_[52]_ after incubation with the respective kinase for 30 min (radiometric assay: mean ± s.d. of duplicate measurements; immunoblot: representative blots of two independent experiments). (*d*) Radiometric kinase assay. Three hepta-repeats with either no or continuous phosphorylation marks or serine to lysine substitution were provided as substrate (mean ± s.d. of triplicate measurements). (*e*) Radiometric kinase assay. Time-dependent phosphorylation of non-modified CTD_[3]_ and K7-CTD_[3]_ peptides (mean ± s.d. of triplicate measurements).

Given the central role of RNA pol II CTD phosphorylation in transcription regulation, we further characterized GST-CTD_[52]_ phosphorylation by Cdk10/CycQ. The RNA pol II CTD is a repetitive element of low complexity, bearing 52 repeats of the seven amino acid consensus sequence Y_1_S_2_P_3_T_4_S_5_P_6_S_7_ with some alterations towards the C-terminus [29]. Site-specific phosphorylation within the CTD hepta-repeats is associated with certain stages of transcription progression [30]. In a radiometric kinase assay, Cdk10/CycQ gradually phosphorylated full-length human GST-CTD_[52]_ in a time course experiment (figure 2*a*). Western blot analysis revealed that Cdk10 phosphorylates Ser2, Ser5 and to a lesser extent also Ser7 sites, but not the Thr4 position within the CTD, indicating promiscuity in CTD substrate specificity (figure 2*b*). The activity of recombinant Cdk10/CycQ towards RNA pol II CTD is significantly lower compared to the known CTD kinases Cdk7 and Cdk9, but higher than Cdk12 and almost equal to DYRK1A (figure 2*c*). Comparison of the phosphorylation site specificity shows that although the overall activity of Cdk10 is significantly lower compared to Cdk7 and Cdk9, it displays a stronger pSer2-specific signal (figure 2*c*).

It has been shown that the transcriptional CDKs 12 and 13 are the most effective for CTD activity *in vitro* upon the presence of a priming phosphorylation at the CTD Ser7 position [31,32]. We used a series of synthetic peptides comprising each three CTD hepta-repeats, which carry a phosphorylation mark at either the Y_1_, S_2_, T_4_, S_5_ or S_7_ position in every repeat (figure 2*d*). We also used a CTD peptide in which Ser7 was replaced by lysine, the most frequent substitution within human CTD repeats towards the distal part of the protein. As expected, Cdk10/CycQ phosphorylated the non-phosphorylated consensus peptide, although the overall activity was lower compared with the GST-CTD_[52]_ substrate. Pre-phosphorylation of the CTD at any position did not improve peptide phosphorylation. In fact, pre-phosphorylated templates prohibited subsequent phosphorylation by Cdk10, whereas the Ser7 to Lys substitution stimulated phosphorylation of the CTD peptide by a factor of four as determined in a time course experiment (figure 2*e*). We tested the preference for Lys7 on a larger CTD fragment using recombinant GST-CTD_[9]_ with nine consensus repeats and compared this to a continuously modified S7 K GST-CTD_[9]_-K7 template. Again, Cdk10 showed a fourfold higher phosphorylation efficacy for GST-CTD_[9]_-K7 compared to consensus GST-CTD_[9]_ (electronic supplementary material, figure S2).

### 2.3. Inhibition of Cdk10 by small-molecule CDK inhibitors

Inhibition of transcriptional CDKs is emerging as an important treatment option in cancer therapies [15,33,34]. Cdk10 is mostly described as a negative regulator of tumour growth [17,21,35–38], but one study reports positive effects of Cdk10 downregulation in colorectal cancer cell lines [39].

**Figure 3.**
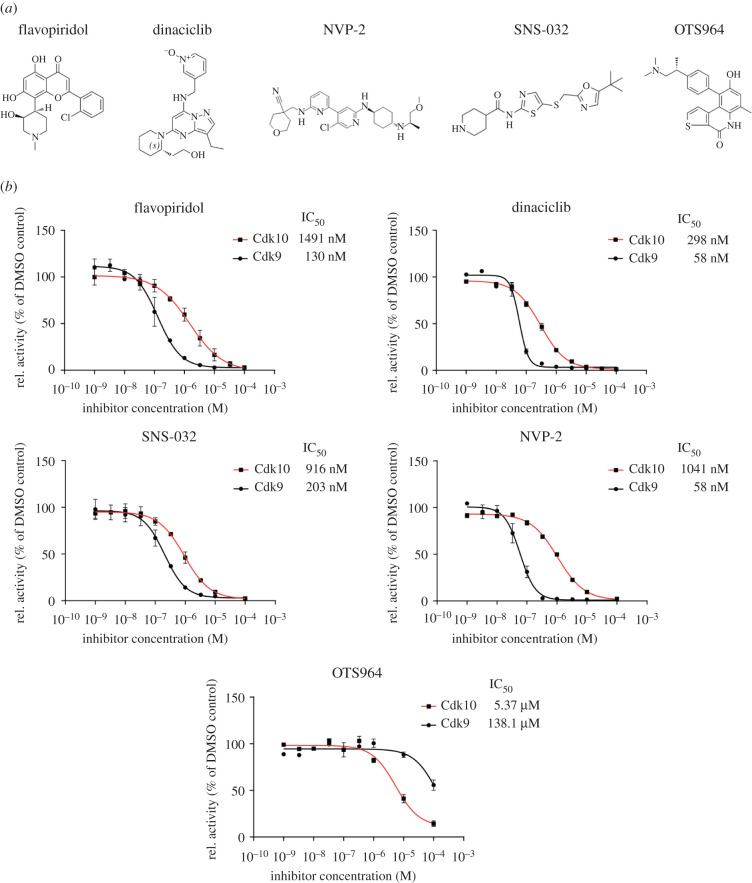
Inhibition of Cdk10 by small molecules. (*a*) Chemical structures of the inhibitors tested against Cdk10/CycQ. (*b*) Radiometric dose–response measurements at 0.2 µM kinase, 50 µM c-MYC (17-167), 1 mM ATP containing 0.35 µCi γ[^32^P]-ATP and indicated concentration of inhibitor (mean ± s.d. of duplicate measurements).

We analysed the inhibitory potential of the ATP-competitive, small-molecule kinase inhibitors flavopiridol, dinaciclib, NVP-2, SNS-032 and OTS964 towards Cdk10 and compared it to Cdk9 (figure 3*a*). Flavopiridol and dinaciclib are well-described inhibitors that potently inhibit a broad spectrum of CDKs including Cdk9 [34]. NVP-2 and SNS-032 have been characterized as potent Cdk9 inhibitors, but kinativ screening in MOLT4 lysates identified binding to Cdk10 [40]. OTS964 was shown to target Cdk11, the closest Cdk10 homologue, in several cancer cell lines [41]. We determined the IC_50_ values of these compounds at the concentrations of 200 nM kinase, 1 mM ATP and 5 mM Mg^2+^ using 50 µM c-MYC as substrate. At these conditions, Cdk10 was inhibited by flavopiridol, dinaciclib, SNS-032 and NVP-2 with IC_50_ values of 1491 nM, 298 nM, 916 nM and 1041 nM, respectively (figure 3*b*). However, these compounds showed a significantly higher potency towards Cdk9 inhibition with IC_50_ values of 130 nM, 58 nM, 203 nM and 58 nM, respectively. By contrast, the Cdk11 inhibitor OTS964 displayed the weakest Cdk10 inhibition among the tested compounds with an IC_50_ of 5.37 µM but was the only one which displayed significant advantage over Cdk9 (IC_50_ of 138.1 µM). We also tested the FDA-approved Cdk4/6 inhibitors abemaciclib and palbociclib and the pre-clinical covalent Cdk7/12/13 inhibitors THZ-1 (Cdk7/12/13), YKL-5-124 (Cdk7) and THZ-531 (Cdk12/13) for potential cross reactivity with Cdk10. At conditions that result in full inhibition of their primary target, we did not observe any considerable effect of these inhibitors on Cdk10 (electronic supplementary material, figure S3). Whereas the pan-CDK and the Cdk9/10 inhibitors showed markedly better inhibition of Cdk9, the Cdk11 inhibitor OTS964 might provide a scaffold for the development of Cdk10 selective compounds that do not cross-react with Cdk9.

### 2.4. Identification of Cdk10 substrates by chemical genetics coupled to mass spectrometry

**Figure 4.**
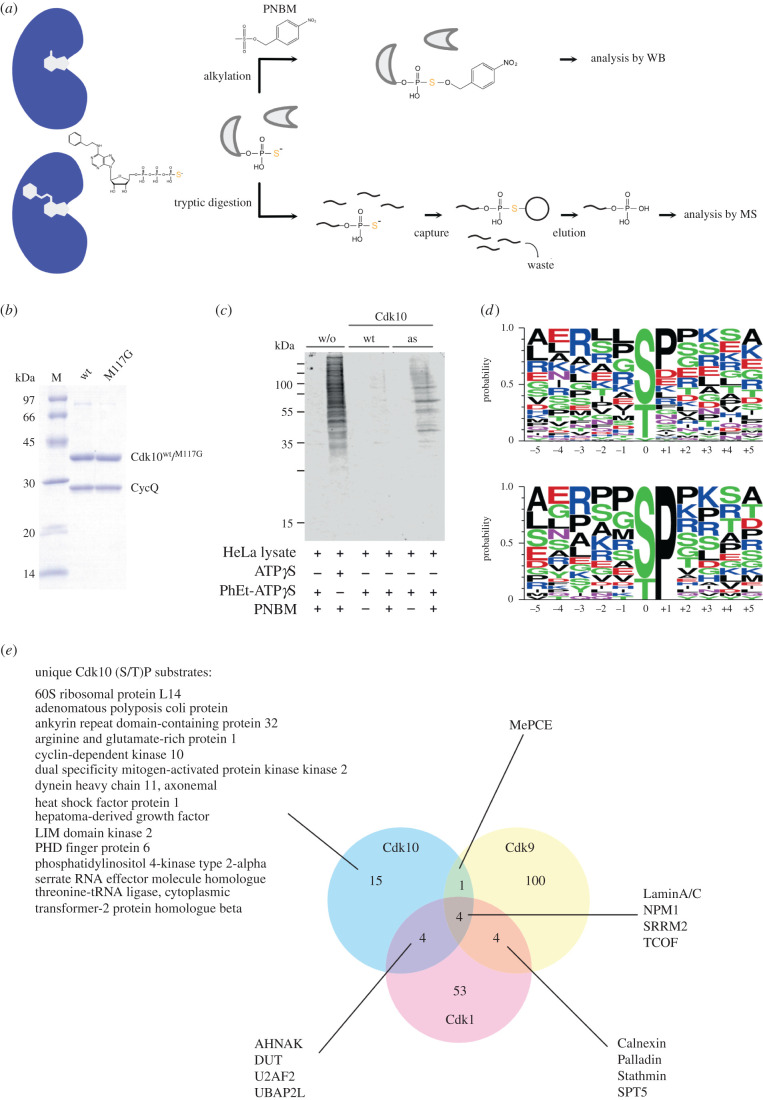
A chemical genetic screen identifies novel protein substrates of Cdk10/CycQ. (*a*) Schematic of the identification of Cdk10/CycQ substrates. Samples were thio-phosphorylated by the addition of Cdk10^as^/CycQ and PhEt-ATPγS. As control, lysates were incubated with Cdk10^wt^/CycQ. Proteins were digested by trypsin and thio-phosphorylated peptides were captured with iodo-acetyl beads. After elution, peptides were analysed by mass spectrometry. In parallel, an aliquot of the sample was alkylated with para-nitrobenzylmesylate (PNBM) and analysed by immunoblot to monitor labelling efficiency. (*b*) SDS–PAGE analysis (Coomassie) of 2 µg wild-type Cdk10/CycQ and analogue-sensitive Cdk10M117G/CycQ complexes. (*c*) Immunoblot analysis of thio-phosphorylated proteins in HeLa cell lysate. (*d*) Consensus sequence of the Cdk10 phosphosites deduced from the dataset. Upper panel: all phosphosites; lower panel: restricted to phosphosites followed by a proline. (*e*) Comparison of (S/T)P site protein substrates of the kinases Cdk10/CycQ (this paper), Cdk1/CycB1 [42] and Cdk9/CycT1 [43].

To get insights into Cdk10-mediated cellular pathways, we set up an *in vitro* chemical genetic screen for the identification of Cdk10/CycQ substrates by mass spectrometry following an approach that was developed for the identification of Cdk1/Cyclin B substrates [42]. For this screen, we introduced a point mutation (M117G) into the Cdk10 ATP-binding pocket. The Cdk10 (M117G) mutant is able to use N^6^-modified bulky ATP analogues as substrates due to the enlargement of the ATP-binding site. These ATP analogues are not used by any native kinase allowing specific labelling of proteins in cell lysates (figure 4*a*). To discriminate Cdk10 substrates from other phosphoproteins in the cell lysate, ATPγS variants of the analogues are used. The transfer of the terminal biorthogonal thio-phosphate to the substrates assists in monitoring of the labelling reaction by western blot analysis using a specific antibody. In addition, the thio-phosphorylation can be detected by mass spectrometry analysis of the purified thio-phosphorylated peptides via iodo-acetyl beads by a covalent capture and release mechanism (figure 4*a*).

We expressed and purified the analogue-sensitive Cdk10(M117G)/CycQ (Cdk10^as^/CycQ) complex to homogeneity (figure 4*b*) following the protocol established for the wild-type proteins. The Cdk10^as^/CycQ complex retained activity at comparable levels to wild-type Cdk10 but displayed a higher *K*_m_ value for ATP (electronic supplementary material, figure S4). Testing of different commercially available ATPγS analogues confirmed analogue sensitivity of the Cdk10 (M117G) mutant and identified N^6^-phenylethyl-ATPγS (PhEt-ATPγS) as the best-suited analogue for Cdk10^as^/CycQ (electronic supplementary material, figures S4D and S4E). We next assessed if PhEt-ATPγS can be used to specifically thio-phosphorylate proteins in the cell lysate. HeLa full-cell lysate was incubated with PhEt-ATPγS or ATPγS and the resulting thio-phosphorylation was monitored after alkylation of the sulfur with para-nitrobenzylmesylate (PNBM) by western blot. In the absence of any recombinant kinase the addition of PhEt-ATPγS resulted in no thio-phosphorylation of proteins in the cell lysate demonstrating the inability of native kinases to use N^6^-phenylethyl-ATPγS as a substrate (figure 4*c*, lane 1). By contrast, the addition of ATPγS results in a strong signal dispersed throughout the entire lane, validating the use of the thio-phosphate for substrate identification (lane 2). The addition of PhEt-ATPγS to Cdk10^wt^/CycQ showed only a faint signal of phosphorylation compared to the sample in the absence of recombinant kinase (lanes 3 and 4). Recombinant Cdk10^as^/CycQ in contrast was considerably more potent in thio-phosphorylating proteins in cell lysate (lanes 5 and 6). Proteins in cell lysates are thus specifically thio-phosphorylated by Cdk10^as^/CycQ with a good signal to noise ratio.

### 2.5. Cdk10 phosphorylates RNA regulation, transcription and translation factors

For the identification of Cdk10 substrates from cell lysates, we thio-phosphorylated proteins in HEK293 full-cell lysate and in nuclear extracts. Identified proteins were considered as Cdk10 substrates if they were found in the Cdk10^as^ sample but not in the Cdk10^wt^ control. For proteins which were identified in both Cdk10^wt^ and Cdk10^as^, peptides which were at least twofold enriched in the Cdk10^as^ treated sample over the Cdk10^wt^ control were considered as Cdk10 substrates. Using this threshold, we identified 89 different Cdk10 phosphorylation sites, originating from 66 different proteins (electronic supplementary material, table S1). Almost all of the phosphosites found in our screen have been detected before in phospho-proteomic studies to occur *in vivo* and are deposited in proteomic databases, validating our analogue-sensitive screening assay. The individual datasets are shown in electronic supplementary material, table S1.

We identified proteins involved in mRNA transcription (MePCE, TRIM28), splicing (U2AF2, THRAP, SRRM2), nuclear scaffold (SRRM2, LAMIN A/C), miRNA processing (SRRT), translation (EEF1D, EIF3D, EIF4B, RPL14, RPL5), rRNA regulation (PHF6, TCOF1, NOLC), cell cycle regulation (NPM1, ZC3HC, ANKRD17), growth factor signalling (HDGF, ARGLU1, NCOA7), stress response (HSF1, UBAP2 L, HSPB1) and kinase signalling (LIMK2, MAP3K5, PI4K2A, MAP2K2, Cdk10). Analysis of GO-term enrichment of our dataset compared to the entire human proteome showed that Cdk10 substrates were indeed enriched for RNA regulation, transcription and translation-related processes (electronic supplementary material, figure S5). These data elucidate widespread cellular functions of Cdk10.

The dataset comprising 89 different phosphorylation sites was used to determine a Cdk10 substrate consensus sequence, revealing a preference for an (S/T)P motif, which is regarded as the minimal consensus sequence of CDKs within in CMGC branch of the kinome. However, apart from a preference for proline in the +1 position no apparent consensus N- or C-terminal of the phosphorylation site can be identified (figure 4*d*). In a second step, we restricted the analysis to (S/T)P motifs, which resulted in 24 remaining phosphosites. Now, positively charged amino acids lysine (K) and arginine (R) in the +3 position become more prominent such that 35% of (S/T)P peptides resemble the consensus SPx(K/R) motif of CDKs and MAP kinases (figure 4*d*). Moreover, 25% of (S/T)P peptides contained a positively charged residue in the +2 position.

We compared our data analysis with previous studies using the same technique to identify substrates of Cdk9/CycT1 [43] or Cdk1/CycB [42], respectively (figure 4*e*). For this comparison, we focused on the (S/T)P substrates. Our Cdk10/CycQ dataset contained 24 different putative (S/T)P substrates, the screen for Cdk9/CycT1 substrates identified 109 different proteins, whereas 65 different proteins were identified for Cdk1/CycB. Of the 24 (S/T)P containing proteins, nine are shared with Cdk9 or Cdk1, leaving a remainder of 15 proteins that can be assigned to be uniquely phosphorylated by Cdk10. Cdk10 shares four substrate proteins with Cdk1 (AHNAK, DUT, U2AF2, Ubap2 L) and one (MePCE) with Cdk9. The remaining four proteins (LaminA/C, NPM1, SRRM2, TCOF) were identified in all three datasets. Of note, aside from these targets, Cdk1 and Cdk9 have four substrates (Calnexin, Palladin, Stathmin, SPT5) in common. The low overlap of substrates appears more pronounced when comparing actual phosphosites within the proteins. When restricted to phosphorylation sites, only TCOF Ser156 remains as common substrate site among all three kinases (electronic supplementary material, figure S5).

### 2.6. *In vitro* validation of Cdk10 substrates

We selected eight Cdk10 substrates from our analogue-sensitive kinase screen that are representative for cellular processes Cdk10 might be involved in, such as transcription (TRIM28), splicing (U2AF2), cell cycle (NPM1), stress response (UBAP2 L, HSF1), microRNA processing (SRRT), kinase signalling (LIMK2) and growth factor response (HDGF) to analyse their phosphorylation by Cdk10/CycQ with recombinant proteins *in vitro*.

**Figure 5.**
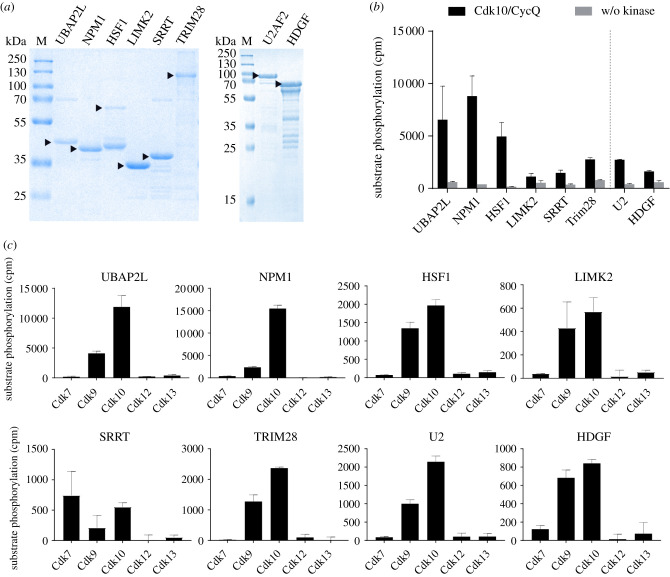
*In vitro* validation of Cdk10 substrates. (*a*) SDS–PAGE analysis (Coomassie) of UBAP2L (355–652), NPM1 (isoform2), MBP-HSF1 (271–384), LIMK2 (149–242), SRRT (409–602), GST-TRIM28 (1–835), GST-U2AF2 (2-475, isoform2), GST-HDGF (1–240), 2 µg each (arrowheads); kDa, kilodalton; M, marker. (*b*) Radiometric kinase assay. Substrates at a concentration of 25 µM (UBAP2L, NPM1, SRRT, LIMK2, U2, HDGF) 15 µM (HSF1), 10 µM (TRIM28) were incubated with 0.2 µM Cdk10/CycQ for 1 h at 30°C. U2 and HDGF were measured separately from the other samples, indicated by the dashed line (mean ± s.d. of duplicate measurement). (*c*) Radiometric kinase assay. The same as in (*b*), except that the background of the w/o kinase control was subtracted for reasons of clarity (mean ± s.d. of duplicate measurement).

For substrate validation, the proteins or domains containing the phosphorylation site were purified from expression in *Escherichia coli* cells (figure 5*a*, arrowheads). Phosphorylation was analysed in a radioactive kinase assay by incubation with 0.2 µM Cdk10/CycQ and 1 mM ATP for 1 h at 30°C. As control, proteins were incubated in absence of kinase. All tested proteins were exposed to phosphorylation by Cdk10/CycQ (figure 5*b*). Analysis by mass spectrometry confirmed substrate phosphorylation and, in some cases, revealed additional phosphorylation sites within the recombinant proteins compared to the screening (electronic supplementary material, figure S6 and table S2). The only exception was NPM1, which was exclusively phosphorylated at Thr237 but not Ser70 using recombinant protein, in contrast to the results from crude extracts. Of note, mass spectrometry confirmed phosphorylation of TRIM28 Ser19, which was also detected in crude extracts but narrowly missed our threshold criterion of twofold enrichment. TRIM28 Ser19 thus probably represents a common target of Cdk9 and Cdk10.

To address potential substrate promiscuity among CDKs we probed the substrates with the related kinases Cdk 7, 9, 12 and 13. In fact, Cdk9 was capable to phosphorylate all of the Cdk10 substrates *in vitro* and SRRT was phosphorylated by Cdk7, Cdk9 and Cdk10. However, except for SRRT, Cdk10 displayed the highest activity towards the identified substrates in all cases (figure 5*c*).

### 2.7. Cdk10 is an *in vitro* substrate of Cdk1 and Cdk5

**Figure 6.**
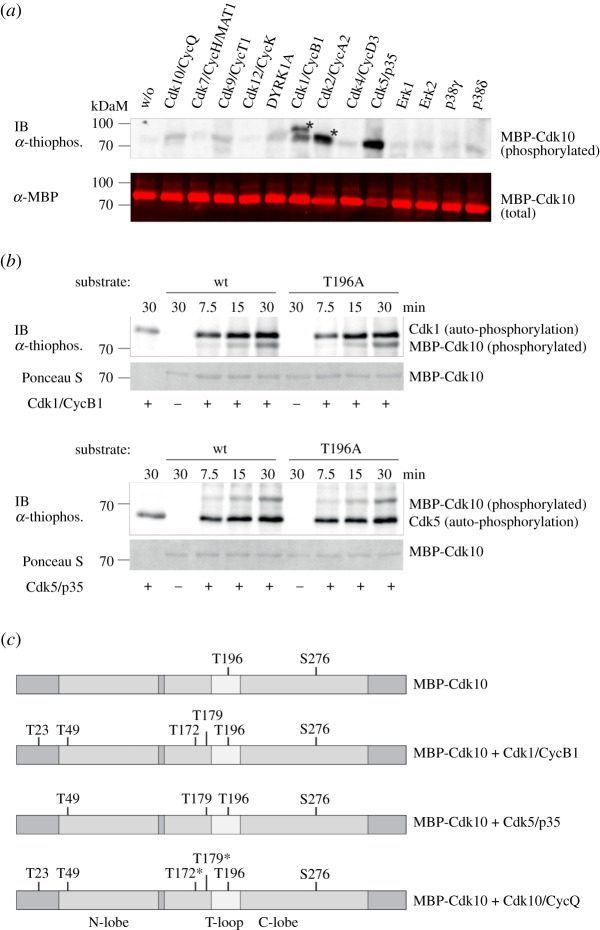
CDK cross-talk to regulate Cdk10 activity. (*a*) Immunoblot analysis of thio-phosphorylation (upper panel) or MBP (lower panel) of 1 µg MBP-Cdk10 after incubation with 0.2 µM of the indicated kinase for 60 min. Asterisks indicate GST-CycB1/GST-Cdk1 and GST-CycA2/GST-Cdk2 autophosphorylation. (*b*) Immunoblot analysis of thio-phosphorylation of 1 µg MBP-Cdk10 and MBP-Cdk10T196A after incubation with 0.05 µM Cdk1/CycB1 or Cdk5/p35NCK. Below, Ponceau S staining of the membrane. (*c*) Results of the mass spectrometric determination of phosphorylation sites of MBP-Cdk10 after 20 min incubation without any additional kinase or after incubation with Cdk1/CycB1, Cdk5/p35 or Cdk10/CycQ. The asterisk indicates phosphosites that were identified only after longer incubation times of 40 or 120 min.

We probed a set of several proline-directed kinases from the CMGC branch, namely Cdk9/CycT1, Cdk12/CycK, DYRK1A, Cdk1/CycB1, Cdk2/CycA2, Cdk5/p35, Erk1, Erk2, p38γ, p38δ for their ability to phosphorylate MBP-Cdk10 *in vitro* (figure 6*a*). Among the tested kinases, Cdk1/CycB1 and Cdk5/p35 displayed the strongest signals providing an interesting option for Cdk cross-talk at the two main roles Cdk10 has been described for, cell cycle G2/M phase and neural development. However, using the Cdk10T196A mutant we did not see any differences in phosphorylation strength, suggesting that Cdk10T196 is not the phosphorylation site of Cdk1 and Cdk5 (figure 6*b*). We used mass spectrometry to map the phosphorylation sites at Cdk10 upon incubation with Cdk1 or Cdk5 (figure 6*c*). After 20 min of incubation, we identified four additional phosphorylation sites upon incubation with Cdk1 (T23, T49, T172, T179) and three additional phosphorylation sites after incubation with Cdk5 (T49, T172, T179). Phosphorylation of inactive MBP-Cdk10 by active Cdk10/CycQ for 20 min resulted in only two additional phosphosites (T23, T49), however, longer incubation times (40 and 120 min) resulted in the phosphorylation of Cdk10 Thr172 and Thr179 as well (figure 6*c*). Together, these observations let us conclude that Cdk10 Thr172 and Thr179 are preferentially phosphorylated by Cdk1 and Cdk5.

We also observed Cdk10 autophosphorylation in these experiments (electronic supplementary material, figure S7). This is of particular interest, because in our screening we detected the Cdk10 activation site, T196, as a potential Cdk10 substrate. However, using the MBP-Cdk10T196A mutant we were not able to confirm the auto-activation of recombinant Cdk10. Likewise, we were not able to detect phosphorylation of Cdk10 by Cdk7, the metazoan Cdk-activating kinase [44–50], in our setup. However, these analyses suffer from technical limitations such as the use of ATPγS as co-substrate and cyclin-free Cdk10, as well as the lack of a phospho-specific anti-pT196 Cdk10 antibody. These limitations leave Cdk10 T-loop phosphorylation to be evaluated in future studies.

## 3. Discussion

In this study, we provide a detailed analysis of Cdk10 substrate specificity and function. Using recombinant, full-length Cdk10/CycQ protein complexes including T-loop mutants we show that Cdk10 phosphorylation at Thr196 is dispensable for complex formation but critical for kinase activity. Based on the relationship of Cdk10 to other CDKs, we identify RNA pol II CTD, c-MYC and Rb1 as *in vitro* substrates of Cdk10/CycQ. Further analysis of the CTD phosphorylation pattern revealed that Cdk10 phosphorylates all serine positions within a CTD heptad. Although Cdk10's general activity was lower compared to Cdk9, it displayed a strong site-specific activity for Ser2 within the CTD repeats. However, Cdk10 was unable to phosphorylate CTD repeats that were already phosphorylated at either residue. By contrast, CTD phosphorylation was four-fold increased by the naturally occurring substitution of Ser7 to lysine. In this regard, Cdk10 substrate recognition differs from the related transcription kinases Cdk9, Cdk12 and Cdk13, whose activity was not altered by lysine, but which display increased activity upon Ser7 CTD pre-phosphorylation [31,32,51]. Lysines within the CTD have been shown to contribute to transcriptional regulation by reversible acetylation, which is important for the transcription of epidermal growth factor responsive genes [52].

So far, no low-molecular-weight compound is available that selectively inhibits Cdk10. We determined the IC_50_ values of the Cdk inhibitors flavopiridol, dinaciclib, SNS-032, NVP-2 and OTS964. The pan-selective (flavopiridol, dinaciclib) and Cdk9 selective (SNS-032, NVP2) compounds inhibited Cdk10 activity at low micromolar or even sub-micromolar concentration but were at least 5 times more potent towards Cdk9. The difference in the efficacy of SNS-032 in favour of Cdk9 could explain the specific effect of the SNS-032-based Cdk9 degrader THAL-SNS-032, which degrades Cdk10 only after a longer incubation period [40]. A recent study reported Cdk10 inhibition by these compounds at low nanomolar concentration [53]. This difference presumably results from the use of a 100-fold lower ATP concentration compared to our assay. By contrast to the pan-, and Cdk9 selective Cdk inhibitors, the Cdk11 inhibitor OTS964, albeit showing the least potency for Cdk10 (IC_50_ of 5.38 µM), was the only compound which displayed superior inhibition of Cdk10 over Cdk9 (IC_50_ of 138.1 µM). OTS964 might thus provide a scaffold for the development of selective Cdk10 inhibitors.

Until now, only two protein substrates of Cdk10 have been confirmed: ETS-2 and PKN2 [5,23]. With our dataset, we significantly expand the knowledge of the protein substrate repertoire of Cdk10 and provide a starting point for further elucidation of Cdk10 function. We identified diverse substrates, among which some were involved in transcription, translation, cell cycle, heat shock response and growth factor signalling. However, we identified neither ETS-2 nor PKN2 in our screen. The enrichment of thio-phosphopeptides is based on sulfur binding to iodo-acetyl. Hence, cysteines can prevent the enrichment of thio-phosphorylated peptides by binding to the affinity matrix via the cysteine. In fact, when establishing the thio-enrichment procedure we experienced difficulties to enrich the cysteine-rich recombinant c-MYC (17-167) despite good thio-phosphorylation *in vitro* (data not shown). Both ETS-2 and PKN2 contain cysteines in close vicinity to the described Cdk10 phosphorylation site, which could have prevented their identification.

From our dataset, we derived a Cdk10 consensus sequence for substrate recognition which revealed a preference for SP motifs. This is in accordance with (S/T)P being the minimal consensus sequence of CDKs and MAPKs. Nonetheless, only 35% of the identified substrate sites contained this motif. When we restricted our analysis to (S/T)P phosphorylation sites, a preference for positively charged amino acids at positions −3, +2 and +3 became visible. Interestingly, this is consistent with the Cdk10/CycQ consensus sequence of SP(K/R)R determined in a systematic peptide array [53]. However, the proline in +1 position was mandatory for efficient phosphorylation of peptides by Cdk10/CycQ whereas the majority of identified phosphosites in our screen were not followed by proline. We assume that this difference reflects the more complex sequence recognition of protein substrates compared to peptides. Of note, in our mass spectrometric analysis of recombinant Cdk10 protein substrates, we identified several phosphorylation sites which were not followed by proline, underscoring that Cdk10/CycQ is not strictly proline directed.

Cdk10 has been found to be important for neural development in different species, including humans [16,17,54]. The strong developmental phenotypes are contrasted by the lack of any apparent impairment upon Cdk10 interference in cultured cells. However, growth factor deprivation results in elongated cilia upon siRNA mediated Cdk10 knock-down and in Cdk10 KO cells, which suggests a growth factor specific activity of Cdk10 [16,23]. The observation that Cdk10 downregulation can cause resistance to tamoxifen, which is an antagonist of the female growth hormone oestrogen, further supports this idea [21]. Likewise, the insect steroid growth hormone ecdysone was shown to activate the lepidopteran Cdk10 homologue, resulting in altered transcription signatures [55]. In fact, we identified hepatoma-derived growth factor (HDGF) and arginine-glutamate rich protein 1 (ARGLU1) as direct Cdk10 substrates. Phosphorylation of HDGF at Ser165 is required for the processing and secretion of HDGF [56]. ARGLU1 has been described to mediate oestrogen receptor-dependent gene transcription [57] and a recent study identified ARGLU1 as a transcriptional co-activator upon glucocorticoid receptor stimulation [58]. Intriguingly, the study reports Cdk10 and Cyclin Q as transient ARGLU1 interaction partners in bio-ID mass spectrometry experiments, further supporting a role of Cdk10/CycQ in oestrogen and glucocorticoid hormone-mediated gene transcription.

Enzymatic activity of Cdk10/CycQ depends on phosphorylation of the conserved Cdk10T196 in the T-loop. We found that Cdk1/CycB1 and Cdk5/p35 phosphorylate Cdk10 at several sites within and outside the kinase domain, but not at T196, offering the possibility of cross-talk between these kinases during the cell cycle and neural development. An interesting, yet unconfirmed finding is a potential Cdk10 auto-activation, which needs to be addressed in future studies.

## 4. Opening up

CDKs are currently grossly grouped into those driving the cell cycle and those mediating transcription [1]. Although they comprise a good working model, highlighting the main function of these kinases, we hypothesize that these categories will be challenged in the future. There are already vital examples of CDKs acting in both the cell cycle and transcription with Cdk7 serving as CAK and TFIIH subunit being the most prominent one [59]. Also, Cdk11, which plays a role in mRNA splicing, has been shown to affect mitosis via coordination of the mitotic spindle [60]. Moreover, a recent study uncovered a direct transcriptional effect of the G1 cdk-cyclin pair Cln3/Cdk1 [61]. We expect more of such interdisciplinarity and interconnectivity to be discovered among CDKs. When comparing datasets of Cdk1 and Cdk9 substrates in this paper we noted SPT5 Ser666 as a shared substrate. Phosphorylation of SPT5 at this site has been shown to contribute to transcription regulation by Cdk9 [62], and thus offers an additional common trait of cell cycle and transcriptional CDKs.

Based on our data and previous studies, we speculate that Cdk10 obtains a hybrid position between cell cycle and transcriptional CDKs, and acts as an integrator of external and internal stimuli such as cell stress, growth factors or DNA damage. With its ability to directly phosphorylate the RNA pol II CTD but also factors which affect growth factor release (HDGF) or modulation of growth factor responses (ARGLU1), the stress response (HSF1, UBAP2L), cell cycle progression (NIPA, NPM1), rRNA synthesis (PHF6, TCOF, NOLC) and translation (RPL14), the Cdk10/CycQ complex is well suited to centrally coordinate these processes upon stimulation.

## 5. Experimental procedures

### 5.1. Cloning, generation of multi-gene-expression vectors

All expression plasmids were generated by restriction enzyme-based cloning (electronic supplementary material, table S3). Restriction enzyme cleavage sites were introduced by PCR. All plasmid inserts were confirmed by Sanger sequencing. Plasmids containing multiple genes for co-expression in *Sf9* insect cells were generated using the multibacTurbo system using pACEBac1 vectors modified in house with N-terminal MBP or GST affinity tags. The vectors were fused with pIDK and pIDC donor vectors by Cre-recombination. Successful Cre-recombination was validated by antibiotic selection and the stoichiometry of the fusion vector confirmed by restriction enzyme digest.

### 5.2. Site-directed mutagenesis

Point mutations were introduced into plasmids by primer directed mutagenesis in a PCR reaction (electronic supplementary material, table S3). The non-mutated parental plasmids were digested by adding 5 µl 10× cutsmart buffer (NEB), and 1 µl *Dpn*I (NEB) for 1 h at 37°C directly to the PCR reaction mixture after PCR. After restriction digest, 5–10 µl of the reaction mixture were used to transform NEB β10 cells.

### 5.3. Transfection of *Sf9* cells and virus propagation

*Sf*9 cells were transfected with bacmid DNA by lipofection using Cellfectin (Invitrogen). Transfection was performed in 6-well format with 2 ml (0.35 × 10^6^ cells ml^−1^) *Sf9* cells per well. For transfection, 10 µl of Bacmid DNA was mixed with 100 µl serum-free medium. In a different tube 8 µl Cellfectin was mixed with 100 µl medium. Both solutions were mixed and incubated for 15–30 min to allow the formation of DNA–lipid complexes. After incubation, 200 µl were added to the respective well. Cells were incubated for 72 h at 27°C. After 72 h the virus containing supernatant was collected (V_0_) and sterile filtered. V_0_ was then used for virus amplification.

V_0_ was used to infect 50 ml of *Sf*9 cells (0.5 × 10^6^ cells ml^−1^). Successful infection of the *Sf9* cells was monitored by a stop of cell division of transfected cells due to viral infection. Therefore, cells were counted every day and adjusted to 0.5 × 10^6^ cells ml^−1^ (50 ml) until the cells stopped dividing. After replication has stopped, cells were incubated for another 48 h and then centrifuged at 500 r.p.m. for 20 min. The supernatant containing the virus was collected (V_1_). To obtain higher-titre virus (V_2_), 1 ml of V_1_ was used to infect 100 ml *Sf*9 cells (1 × 10^6^ cells ml^−1^). Cells were incubated for four days and afterwards centrifuged at 500 r.p.m. for 20 min, and supernatant was collected (V_2_). All viral stocks were sterile filtered and stored at 4°C.

### 5.4. Protein expression and purification

Proteins were expressed either in *Sf9* insect cells (Cdk10/CycQ, Cdk7/CycH/MAT1, Cdk9, Cdk12/CycK) or *E. coli* BL21 pLysS cells (His-DYRK1A (127–485), GST-CTD_[52]_, GST-CTD_[9]_, GST-CTD_[9]_K7, His-c-MYC (17–167), Rb1 (761–928), GST-SRSF7, GST-UBAP2L, GST-Trim28, MBP-HSF1, His-NPM1, GST-SRRT, GST-U2AF2, GST-HDGF, GST-LIMK2).

For expression in *Sf9* insect cells, cells at a density of 1.5 × 10^6^ cells ml^−1^ were infected by adding 2% (v/v) of baculovirus V_2_ preparation. Cells were harvested after 72 h by centrifugation at 2000 r.p.m. (JLA8.1 rotor, Beckman-Coulter), washed carefully with PBS, snap frozen in liquid nitrogen, and stored at −20°C or −80°C.

For expression of proteins in bacteria, *E. coli* cells were grown in LB medium containing appropriate antibiotics at 37°C (pre-culture). The next day, optical density at 600 nm (OD_600_) was determined and the pre-culture was diluted into larger volumes of LB medium to an OD_600_ of 0.1. Cultures were grown to optical densities of 0.8–1.2 at 37°C for induction of expression. Protein expression was induced by adding IPTG to a final concentration of 0.1–0.5 mM, and the expression temperature was set to 18–37°C.

To harvest bacteria, cells were collected in 1 l buckets by centrifugation at 5000 r.p.m. (JLA.8.1 rotor, Beckman-Coulter) for 20 min. Cell pellets were resuspended in PBS and pelleted again by centrifugation. Bacterial pellets were subjected to cell lysis or snap frozen in liquid nitrogen and stored at −20°C for later use.

### 5.5. Protein purification

For purification cell pellets were resuspended in the respective lysis buffer and lysed by sonication. Cell debris was removed by centrifugation at 20 000–25 000 r.p.m. in a JA250.50 rotor for 30 min at 4°C. The lysate was afterwards filtered through syringe filter with a 0.45 µm pore size. Filtered lysate was then used for affinity chromatography. The only exception was His-c-MYC (17–167), which was purified from inclusion bodies as described below.

### 5.6. Cdk10/CycQ

The coding sequences of human Cdk10 and human Cyclin Q were purchased as synthetic genes (GeneArt, Regensburg). Cdk10 was cloned into a pACEBac1 vector modified with an N-terminal maltose-binding protein (MBP)-tag followed by a tobacco etch virus (TEV) cleavage site. CycQ was cloned into a pIDK vector. A hexahistidine-tag followed by a TEV cleavage site was inserted 5′ of the CycQ sequence by PCR. For co-expression, vectors were fused by Cre recombinase. Cdk10/CycQ complexes were purified by sequential affinity chromatography using pre-packed HisTrap crude FF and MBPTrap HP columns. After equilibration of the affinity columns with lysis buffer (50 mM Hepes, pH7.6, 300 mM NaCl, 20 mM Imidazole, 5 mM β-mercaptoethanol), the lysate was loaded onto a HisTrap column using an ÄKTA prime FPLC system. The column was washed with wash buffer (50 mM Hepes pH 7.6, 300 mM NaCl, 40 mM imidazole, 5 mM β-ME) until UV baseline was reached. The protein was eluted from the column with 250 mM imidazole directly onto a MBPTrap column and the flowthrough was discarded. MBP-Cdk10/His-CycQ complexes were eluted from the MBPTrap column with MBP elution buffer (20 mM Hepes pH7.6, 300 mM NaCl, 10 mM Maltose, 1 mM TCEP). MBP-tag and His-tag were removed by TEV protease digest o/n at 4°C, and the complex further purified by size exclusion chromatography at a Superdex 75 pg column with SEC buffer (20 mM Hepes, 300 mM NaCl, 1 mM TCEP, 10% glycerol).

For co-purification experiments using MBP-Cdk10/His-CycQ, MBP-Cdk10T196A/His-CycQ and MBP-Cdk10T196E/His-CycQ and for purification of non-complexed MBP-Cdk10, MBP-Cdk10T196A, MBP-Cdk10T133A 1 ml pre-packed MBPTrap columns were used at a ÄKTA start FPLC system. Proteins were purified using the following buffers: Lysis/wash buffer: 50 mM Hepes pH7.6, 300 mM NaCl, 1 mM TCEP. Elution buffer: 20 mM Hepes pH7.6, 300 mM NaCl, 10 mM Maltose, 1 mM TCEP.

### 5.7. Cdk7/CycH/MAT1 (CAK)

Human GST-Cdk7 (2–346)/Cyclin H (1–323) was expressed from a single vector using the multibac^turbo^ system. A C-terminal construct of MAT1 fused to an N-terminal maltose-binding protein (MBP-MAT1 230–309) was expressed separately from Cdk7/CycH in Sf9 cells. Cells were lysed in lysis buffer (50 mM Hepes pH7.6, 150 mM NaCl, 5 mM β-mercaptoethanol) For purification of ternary CAK complexes, cell lysates of the individual expressions (GST-Cdk7/CycH and MBP-MAT1 230–309) were pooled prior to purification. Complexes were affinity purified using MBPTrap columns and the columns were washed with lysis buffer until baseline. Proteins were eluted with lysis buffer containing 10 mM maltose. GST and MBP tags were removed by TEV protease digest o/n at 4°C and the complex further purified by size exclusion chromatography on a Superdex 200 pg column and reverse MBP and GST affinity chromatography.

### 5.8. Cdk9/CycT1 (P-TEFb), Cdk12/CycK and Cdk13/CycK

P-TEFb was reconstituted from His-Cdk9 expressed in Sf9 cells and GST-CycT1 (1–272) expressed in *E. coli* prior to affinity purification. P-TEFb was purified as described [51].

GST-CDK12 (714–1063)/GST-Cyclin K (1–267) were co-expressed with CAK1 (*Saccharomyces cerevisiae*) and purified as described [31]. GST-CDK13 (694–1093)/GST-Cyclin K (1–267) were co-expressed with CAK1 (*S. cerevisiae*) and purified as described [32].

### 5.9. DYRK1A

A plasmid of human DYRK1A (127–485) for bacterial expression in a pNIC28-Bsa4 vector was purchased from AddGene (Plasmid no. 38913). DYRK1A was expressed in *E. coli* BL 21 DE3 pLysS cells for 16 h at 18°C after induction at an OD 0.5 with 1 mM IPTG. Cells were lysed in 50 mM Hepes, pH7.6, 500 mM NaCl, 5% glycerol, 0.5 mM TCEP, 5 mM imidazole. His-DYRK1a was purified using HisTrap crude columns. Columns were washed with lysis buffer containing 25 mM Imidazole and the protein eluted with 250 mM Imidazole. His-tag was removed by TEV protease digest and the protein further purified by size exclusion chromatography on a Superdex 75 pg column.

### 5.10. RNA pol II CTD

Full-length human RNA pol II C-terminal domain (GST-CTD_[52]_) was expressed with an N-terminal GST-tag for 16 h at 18°C after induction by 0.1 mM IPTG at an OD_600_ of 0.8. For purification, cells were lysed in lysis buffer (50 mM Hepes pH 7.6, 150 mM NaCl, 5 mM β-ME) by sonication. The lysate was cleared from cell debris by centrifugation at 20 000 r.p.m. in a JA25.50 rotor (Beckman-Coulter) and filtration through a PE filter with 0.45 µm pore size. GST-CTD_[52]_ was affinity purified using GSTrap columns (GE Healthcare). The column was washed with lysis buffer until baseline was reached. After elution in lysis buffer containing 10 mM GSH, the sample was concentrated and subjected to size exclusion chromatography on a Superdex 200 pg column equilibrated in 50 mM Hepes (pH 7.6), 150 mM NaCl, 10% glycerol, 5 mM β-mercaptoethanol. GST-CTD_[9]_ and GST-CTD_[9]_K7 were expressed for 4 h at 30°C after induction with 0.5 mM IPTG at an OD_600_ of 0.6–1.2. GST-CTD_[9]_ and GST-CTD_[9]_K7 were essentially purified as described above for full-length GST-CTD_[52]_ with the exception that gel filtration was performed with a Superdex 75 pg column.

### 5.11. His-c-MYC (17–167)

His-c-MYC (17–167) was expressed for 4 h at 30°C after induction at an OD_600_ of 0.8 with 0.5 mM IPTG. For purification from inclusion bodies, the cell pellet was resuspended in lysis buffer (50 mM Hepes, pH7.6, 100 mM NaCl, 5 mM β-ME) and sonified. The insoluble fraction was collected by centrifugation at 20 000 r.p.m. for 20 min. The supernatant was discarded and the pellet resuspended with wash buffer (50 mM Hepes pH 7.6, 100 mM NaCl 1 M Urea, 0.5% Triton), followed by centrifugation. This step was repeated two times. After washing, the pellet was resuspended in lysis buffer to remove Triton from the sample. Inclusion bodies were pelleted by centrifugation at 20 000 r.p.m. for 20 min. For lysis, the pellet was resuspended in denaturing buffer (50 mM Hepes, pH7.6, 100 mM NaCl, 8 M Urea, 5 mM β-ME) and incubated for 60 min at room temperature. The sample was cleared from non-lysed particles by centrifugation at 25 000 r.p.m. for 30 min at 25°C. The sample was afterwards dialysed into refolding buffer 1 (50 mM Hepes, pH7.6, 100 mM NaCl, 4 M Urea, 5 mM β-ME) for 16 h at room temperature. The next day the sample was dialysed against 50 mM Hepes, pH7.6, 100 mM NaCl, 10 mM imidazole, 5 mM β-ME for 6 h at 4°C. Precipitated protein was removed by centrifugation at 5000 r.p.m. and the supernatant filtered through a 0.45 µM syringe filter. The sample was then further purified using HisTrap FF crude columns (Cytiva) with an ÄKTA prime FPLC system. After washing with refolding buffer His-MYC (17–167) was eluted with 250 mM imidazole. After elution, the protein was dialyzed into storage buffer (50 mM Hepes, pH7.6, 150 mM NaCl, 1 mM TCEP).

### 5.12. GST-SRSF7 (1–248)

GST-SRSF7 was expressed for 4 h at 30°C after induction with 0.5 mM IPTG. The protein was purified using pre-packed GSTrap columns. Cells were lysed in 50 mM Hepes pH 7.6, 150 mM NaCl, 5 mM β-ME. The column was washed with 50 mM Hepes pH 7.6, 1000 mM NaCl, 5 mM β-ME and the protein afterwards eluted with lysis buffer containing 10 mM GSH.

### 5.13. GST-Rb1 (761–928)

The coding sequence of human wild-type retinoblastoma-associated protein 1 (Rb1, UniProt accession number P06400) was purchased from Addgene (no. 82275). Its C-terminal domain (residues 761–928) was cloned into a pGEX-6P1 expression vector containing an N-terminal GST affinity tag followed by a TEV protease cleavage site using *Bam*HI/*Eco*RI restriction sites. GST-Rb1 was purified as described for GST-SRSF7.

### 5.14. GST-HDGF (1–240)

A HDGF cDNA clone was purchased from Origene (CAT no: RC204148) and subcloned into a pET28a vector backbone modified with an N-terminal GST-tev for purification. GST-HDGF was expressed in *E. coli* BL21 pLysS rosetta cells for 16 h at 18°C after induction with 0.3 mM IPTG. GST-HDGF was purified as described for GST-SRSF7.

### 5.15. GST-U2 (2–471), isoform2

U2 cDNA was cloned from a cDNA library (GenBank accession code: DQ896795). The sequence coding for full-length U2 was amplified by PCR and cloned into a pET28a vector backbone modified with an N-terminal GST-tev for purification. GST-U2 was expressed in *E. coli* BL21 pLysS rosetta cells for 16 h at 18°C after induction with 0.3 mM IPTG. GST-U2 was purified as described for GST-SRSF7.

### 5.16. MBP-HSF1 (271–384)

HSF1 cDNA was cloned from a cDNA library (GenBank accession code: DQ896795). The sequence coding for HSF1 (271–384) was amplified by PCR and cloned into a pET28a vector backbone modified with an N-terminal MBP-tev sequence for purification. MBP-HSF1 (271–384) was expressed in *E. coli* BL21 pLysS Rosetta cells for 4 h at 30°C after induction with 0.3 mM IPTG. MBP-HSF1 was purified as described for GST-SRSF7 with the exception that the elution buffer contained 10 mM Maltose instead of GSH.

### 5.17. GST-UBAP2 L (455–652)

UBAP2 L cDNA was purchased from Origene (RC201371) and the sequence coding for UBAP2 L amino acids 355–652 was amplified by PCR and cloned into a pGEX4T1 vector modified to contain a TEV protease cleavage site. GST-UBAP2 L was expressed for 16 h at 18°C after induction with 0.3 mM IPTG. GST-UBAP2 L was purified as described for GST-SRSF7. After elution, GST was removed by TEV protease cleavage and the sample further purified by size exclusion chromatography. Protein was eluted from a Superdex 75 pg column with 20 mM Hepes pH7.6, 150 mM NaCl, 1 mM TCEP.

### 5.18. GST-SRRT (495–602)

SRRT cDNA spanning the SRRT amino acids (495–602) was purchased as a synthetic construct from GeneArt (ThermoFisher) and cloned into a pET28a vector backbone modified with an N-terminal GST-tev for purification. GST-SRRT was expressed in *E. coli* BL21 pLysS rosetta cells for 16 h at 18°C after induction with 0.3 mM IPTG. GST-SRRT was purified using GSTrap columns as described for SRSF7. GST-SRRT was further purified via size exclusion chromatography on a Superdex 75 pg column.

### 5.19. GST-LIMK2 (149–242)

LIMK2 was cloned from a cDNA library (GenBank accession code: DQ896795). The sequence coding for LIMK2 (149–242) was amplified by PCR and cloned into a pET28a vector backbone modified with an N-terminal GST-tev for purification. GST-LIMK2 was expressed in *E. coli* BL21 pLysS rosetta cells for 16 h at 18°C after induction with 0.5 mM IPTG. GST-LIMK was purified as described for GST-SRRT.

### 5.20. GST-TRIM28 (1–835)

An expression construct of GST-Trim28 in a pGEX-5x Vector was purchased from Addgene (plasmid no. 45570). GST-TRIM28 was expressed in *E. coli* BL21 pLysS rosetta cells for 16 h at 18°C after induction with 0.3 mM IPTG. GST-TRIM28 was purified as described for GST-SRRT with the exception that a Superdex 200 pg column was used for size exclusion chromatography.

### 5.21. His-NPM1 (1–265), isoform 2

NPM1, isoform 2 was cloned from a cDNA library (GenBank accession code: DQ896795). NPM1 was amplified by PCR and cloned into a pProEx-HTa vector. His-NPM1 was expressed in *E. coli* BL21 pLysS Rosetta cells for 16 h at 18°C after induction with 0.3 mM IPTG. Cells were resuspended in lysis buffer (50 mM Hepes, pH7.6, 300 mM NaCl, 20 mM Imidazole, 5 mM β-ME) and loaded onto a HisTrap crude 4 FF column using an ÄKTA prime FPLC system. The column was washed with lysis buffer until baseline. His-NPM1 was eluted with a gradient mixing the lysis buffer with elution buffer containing 250 mM imidazole over 100 ml. Peak fractions were analysed by SDS–PAGE and NPM1 containing fractions were pooled. NPM1 was further purified by size exclusion chromatography on a Superdex 200 pg equilibrated in 20 mM Hepes, pH7.6, 300 mM NaCl, 1 mM TCEP.

### 5.22. RNA polymerase II CTD substrate peptides

For kinase activity analyses, various CTD polypeptides were purchased from Biosyntan (Berlin).

### 5.23. *In vitro* kinase assays

Kinase assays with recombinant proteins were performed in kinase assay buffer (50 mM Hepes pH7.6, 34 mM KCl, 7 mM MgCl_2_, 5 mM β-Glycerophosphate, 2.5 mM DTE). If not indicated otherwise, reactions contained 0.2 µM kinase, were started by the addition of ATP to 1 mM final concentration and incubated at 30°C in a shaking incubator.

For detection of the phosphorylation by a gel shift in SDS–PAGE analysis or immunoblot, the reactions were stopped by adding an equal volume of 2× SDS sample buffer. In all other cases, reactions were quenched by the addition of EDTA (25–50 mM final concentration). For the identification of thio-phosphorylation, reactions were carried out with ATPγS and terminated by adding EDTA to a final concentration of 50 mM. Samples were then alkylated with 2.5 mM PNBM for 30 min at room temperature. Samples were then mixed with SDS sample buffer and analysed by western blot.

For quantitative analysis, radioactive kinase assays were performed. Reactions were done in a total volume of 15 µl per sample. If not indicated otherwise, reactions contained 0.2 µM kinase, were started by addition of ATP to 1 mM containing 0.34 µCi γ[^32^P] (Perkin Elmer) and incubated at 30°C in a shaking incubator. Reactions were stopped by adding EDTA to a final concentration of 50 mM. Samples were spotted onto Amersham Protran nitrocellulose membrane (GE Healthcare) filter sheets. Filters were washed three times for 5 min with PBS to remove free, non-reacted ATP. Samples were transferred to 4 ml liquid scintillation vials and immersed in 2 ml liquid scintillator (UltimaGold). Radioactivity was counted in a Beckman Liquid Scintillation Counter (Beckman-Coulter) for 1 min.

### 5.24. IC_50_ determination of compounds

All compounds were solved at a stock concentration of 10 mM in DMSO and further pre-diluted in DMSO for dose–response measurement. For IC50 determination radioactive kinase assays were run as described above in presence of increasing concentration of inhibitor. For analysis, the data were normalized to the DMSO solvent control and the activity plotted against the inhibitor concentration. IC_50_ values were determined from duplicate measurements by sigmoidal curve fitting with a variable slope using GraphPadPrism, v. 7.05.

### 5.25. Immunoblot

For immunoblotting, proteins were separated by SDS–PAGE and then transferred to a nitrocellulose membrane (Optitran BA-S 85, pore size 0.45 µm, GE Healthcare) using a semi-dry blotting chamber at a constant current of 140 mA/gel for 60 min. After transfer, the membrane was blocked in 5% milk-powder in TBS-T for 1 h at room temperature or at 4°C overnight. Then, the membrane was incubated with primary antibody (pSer2 CTD 1 : 100, pSer5 CTD 1 : 500, pSer7 CTD 1 : 100, pThr4 CTD 1 : 100, thiophospho 1 : 5000). Afterwards, the blot was washed 3 × 5 min in TBS-T and was then incubated with an appropriate secondary antibody (1 : 5000) for 1 h. Membrane was again washed 3 × 5 min in TBS-T. For detection, membranes were immersed with ECL-solution (Sigma Aldrich) for 1 min and then analysed with a CCD camera in a XRSChemDoc system (BioRad).

### 5.26. *In vitro* labelling of proteins in cell lysates

For specific labelling of proteins in cell extracts, N^6^-phenyl-ethyl-ATPγS was used as bulky ATP analogue. For each replicate, 2 mg protein from nuclear extracts were incubated with wild-type Cdk10/Cyclin Q or analogue-sensitive mutant Cdk10^M117G^/Cyclin Q complexes, 1 mM ATP, 0.1 mM PhEt-ATPγS, 10 mM MgCl_2_, 0.2 mg ml^−1^ creatine phospho-kinase, and 40 mM creatine phosphate in a volume of 100 µl. The addition of ATP prevents non-specific use of the PhEt-ATPγS. Creatine phosphate and creatine phospho-kinase function as an ATP regenerating system which was shown to further suppress non-specific labelling [47]. The mixture was incubated for 60 min at 25°C at 350 rpm in a shaking incubator. The reaction was stopped by the addition of 20 µl 0.5 M EDTA per replicate. Samples for mass spectrometry were snap frozen in liquid nitrogen and stored at −80°C. For analysis of the reaction by western blot, a small aliquot was alkylated with 2.5 mM PNBM for 30 min at room temperature and then mixed with 2× SDS sample buffer prior to immunoblot analysis. Membranes were probed with an alkylation specific antibody, generated to detect the PNBM alkylated thio-phosphorylation [63].

### 5.27. Thio-phosphorylated peptides covalent capture

Cell lysates were incubated with three volumes of denaturation buffer (8 M Urea, 10 mM TCEP, 100 mM NH_4_HCO_3_, 2 mM EDTA) and incubated at 55°C for 1 h. Samples were cooled to room temperature and diluted to a final concentration of 2 M urea with 50 mM NH_4_HCO_3_ in H_2_O. 1 M TCEP in H_2_O was added to a final concentration of 10 mM, and proteins were digested with trypsin in a 1 : 25 ratio (w/w) overnight at 37°C. Peptides were purified with an Oasis HLB column according to the manufacturer's instruction and concentrated to near dryness using a SpeecVac. Peptides were dissolved in 20 mM HEPES (pH 7.0) in 50% acetonitrile (v/v) in H_2_O. Ultralink iodo-acetyl resin (Thermo Scientific) was activated with 20 mM HEPES (pH 7.0) in 50% acetonitrile (v/v) in H_2_O. The beads were blocked with 5 mg ml^−1^ BSA in 50% acetonitrile (v/v) in H_2_O for 10 min in the dark. Dissolved peptides were incubated with the prepared resin overnight at room temperature in the dark with gentle rocking. After incubation, the resin with the peptides were loaded into a cartridge and the resin was washed once with 1 ml 50% (v/v) acetonitrile in H_2_O and then with 1 ml H_2_O. To minimize non-specific interactions, the resin was washed successively with 1 ml of H_2_O, 5 M NaCl, and with 50% acetonitrile (v/v) in H_2_O with 5% (v/v) formic acid (FA) and then incubated with 10 mM DTT in H_2_O for 10 min in the column. Finally, the resin was resuspended and incubated with 1 mg ml^−1^ Oxone for 10 min. Oxone-eluted phosphopeptides were immediately desalted with a C18 spin column (Harvard Apparatus) and the eluent was dried in a SpeecVac and stored at −80°C until LC–MS/MS analysis.

### 5.28. In-gel digestion for *in vitro* phosphorylated recombinant substrates

Proteins were separated by SDS–PAGE and stained with Coomassie brilliant blue R250 (0.1% Coomassie R250 in 40% ethanol, 10% acetic acid in H_2_O). After destaining with 10% ethanol, 5% acetic acid in H_2_O the protein band of interest was cut out. Gel slices were reduced with 10 mm DTT for 55 min at 56°C, alkylated with 55 mM iodoacetic acid (IAA) for 20 min at 26°C and digested with endoproteinase trypsin with or without endoproteinase Glu-C overnight at 37°C. Extracted peptides were dried in a SpeecVac and stored at −80°C for LC–MS/MS analysis.

### 5.29. LC–MS/MS analysis and database searching

All samples were analysed on a hybrid quadruple-Obitrap mass spectrometer (Q-Exactive HF, Thermo Scientific), coupled to a nanoflow liquid chromatography system (Ultimate 3000 UHPLC, Dionex, Thermo Scientific). Peptides were pre-concentrated and desalted on a trap column (PepMap, C18, 5 µm, 0.3 × 5 mm, Thermo Scientific) at 10 µl min^−1^ in loading buffer (2% (vol/vol) ACN, 0.1% FA). Phosphopeptides purified from cell lysates were separated on a self-made capillary column (ReproSil-Pur 120 C18-AQ, 1.9 µm, 300 × 0.075 mm, Dr Maisch GmbH) using a 90-min linear gradient from 10% to 36% buffer B (0.1% FA in 80% (v/v) ACN) versus a decreasing concentration of buffer A (0.1% FA) at a flow rate of 300 nl min^−1^. The mass spectrometer was operated in data-dependent acquisition (DDA) mode with a top-20 method using higher-energy collisional dissociation (HCD) with an isolation width of 1.4 *m/z* and an NCE setting of 28%. MS spectra from *m/z* 350 to 1600 were acquired at a resolution setting of 120 000 FWHM at *m/z* 200, and MS/MS spectra at a resolution setting of 30 000. AGC target values and maximum injection times for MS and MS/MS were set to 1 × 10^6^ in 40 ms and 1 × 10^5^ in 128 ms, respectively. Phosphopeptides from *in vitro* phosphorylated recombinant substrates were analysed using a 58-min linear gradient (5–10% B, 0–3 min; 10–42% B, 3–46 min; 42–90% B, 46–46.1 min; 90–90% B, 46.1–52 min; 90–5% B, 52–52.1 min; 5–5% B, 52.1–58 min) on the same Q-Exactive HF mass spectrometer coupled with an Ultimate 3000 RSLC (Dionex). The mass spectrometer was operated in DDA mode using a top 20 method with a survey scan resolution setting of 70 000 FWHM and an MS/MS resolution setting of 17 500 FWHM at 200 *m/z*. HCD was performed with an NCE setting of 30and an isolation width of 2.0 *m/z*. AGC target values and maximum ion injection times for MS and MS/MS were set 1 × 10^6^ in 50 ms and 1 × 10^5^ in 110 ms, respectively. For both, fixed first mass and dynamic exclusion values were set to 110 *m/z* and 25 s, respectively.

The Cdk10 T-loop raw files were processed using MaxQuant software (v. 1.5.5.1 and v. 1.6.5.0, MPI for Biochemistry) [64]. MS/MS spectra were searched against a UniProtKB human database containing 92 954 protein entries (downloaded on February 2017) supplemented with 245 frequently observed contaminants via the Andromeda search engine [65]. Precursor and fragment ion mass tolerances were set to 6 and 20 ppm after initial recalibration, respectively. STY phosphorylation, protein N-terminal acetylation and methionine oxidation were allowed as variable modifications. Enzyme specificity was set to trypsin allowing N-terminal cleavage to proline. Minimal peptide length was set to seven amino acids, with a maximum of two missed cleavages. The false discovery rate (FDR) was set to 1% on the peptide, modification site, and protein level using a forward-and-reverse concatenated decoy database approach.

All raw files for Cdk10 after incubation with Cdk1 and Cdk5, RB1 phosphosites, U2 phosphosites, HDGF phosphosites were processed using Proteome Discoverer (v. 2.5, Thermo Scientific). MS/MS spectra were searched against a fasta database including Cdk10, U2AF2, HDGF and RB. Spectrum files, spectrum selector, Sequest HT and Fixed Value PSM Validator were included in the processing workflow. Precursor mass range was from 350 to 5000 Da. Trypsin plus Glu-c was set for full digestion. Maximum missed cleavage sites was two, and six was set for minimum peptide length. Precursor and fragment mass tolerance were set 10 ppm and 0.02 Da, respectively. S,T,Y phosphorylation, protein N-terminal acetylation and methionine oxidation were allowed as variable modifications. Cysteine carbamidomethylation was set as a fixed modification. In the consensus workflow, seven nodes were used: MSF files, PSM grouper, peptide validator, peptide and protein filter, protein scorer, protein FDR validator and protein grouping. For the validation, protein, PSMs and peptides FDR were set with strict FDR of 0.01 and relaxed FDR of 0.05.

## Data Availability

All of the raw files and search results have been deposited to the ProteomeXchange Consortium via the PRIDE [66] partner repository with the dataset identifier PXD028792. The data are provided in the electronic supplementary material [67].
